# Nursing Students' Perceptions of Instructional Approaches in Nursing Diagnosis Education: A Qualitative Study

**DOI:** 10.1002/nop2.70571

**Published:** 2026-04-26

**Authors:** Sıdıka Kestel, Fatoş Korkmaz

**Affiliations:** ^1^ Fundamentals of Nursing Department, Faculty of Nursing Hacettepe University Ankara Turkey

**Keywords:** NANDA‐I nursing diagnoses, nursing education, nursing students

## Abstract

**Aim:**

This study explored nursing students' perceptions of the instructional approaches to teaching nursing diagnosis.

**Design:**

An exploratory, descriptive qualitative study design was utilized.

**Methods:**

A total of 29 fourth‐year nursing students from eight public universities in Turkey were recruited through social media and snowball sampling. Data were collected via face‐to‐face interviews conducted on Zoom between May and June 2023 and transcribed verbatim. Thematic analysis and constant comparison were used for data analysis. The study adhered to COREQ guidelines.

**Results:**

Thematic analysis identified four main themes with subthemes reflecting students' perspectives: (1) Teaching methods, including (i) inadequate practical training in clinical settings and (ii) ineffective feedback in clinical practice; (2) Utilization of NANDA‐I diagnoses in practice, encompassing (i) challenges in applying NANDA‐I diagnoses and (ii) perceptions of suboptimal implementation by professionals; (3) Recommendations for improving nursing diagnosis education; and (4) Students' perceptions of NANDA‐I diagnoses.

**Conclusion:**

Nursing students perceived current instructional approaches to nursing diagnosis as insufficient, particularly regarding practical training and feedback. Addressing these gaps is crucial for improving the quality of nursing education and practice.

**Implications:**

The findings provide valuable insights for revising nursing curricula to enhance the teaching of nursing diagnosis. Supporting students in mastering nursing diagnoses before graduation is essential for standardizing nursing terminology and improving the quality of care in future practice.

**Reporting Method:**

The COREQ guidelines have been followed.

**Patients or Public Contribution:**

No patients or public contribution.

## 1 Introduction

Most nursing programs incorporate nursing diagnosis as a foundational component of the nursing process (Kume et al. 2016; Tiwaken et al. 2015). Common instructional strategies include lectures, patient care plans, and case‐study discussions. However, significant variability in educational content and methodologies across institutions suggests an ongoing need for refinement in teaching nursing diagnosis (Mousavinasab et al. 2020).

While extant research has addressed challenges associated with determining nursing diagnoses (Ardahan et al. 2019; Basit and Korkmaz 2021; Emine and Asuman 2022) and has explored diverse teaching methodologies (Abbasi et al. 2017; Basit and Korkmaz 2021; Ordu and Çalışkan 2023), limited attention has been given to nursing students' assessments of these approaches. This gap is significant, as students' evaluations provide critical insights into the effectiveness of instructional strategies (Kember et al. 2002). The present study investigates nursing students' perceptions of the pedagogical methods employed in teaching nursing diagnosis to address this gap. Specifically, it examines whether formalized training on North American Nursing Diagnosis Association International (NANDA‐I) nursing diagnoses is provided and identifies the instructional methodologies utilized. The following research questions guide this inquiry:

1. Do undergraduate nursing students receive formalized training on NANDA‐I nursing diagnoses within their academic programs?
2. What instructional methodologies are employed in teaching NANDA‐I nursing diagnoses?

## 2 Background

The North American Nursing Diagnosis Association International (NANDA‐I, 12th edition) has officially catalogued 267 nursing diagnoses, categorized into 13 domains and 47 classes. As the leading authority on defining, researching, revising, disseminating and globally standardizing nursing diagnoses, NANDA‐I is pivotal in shaping nursing diagnostics worldwide (Herdman et al. 2021). In undergraduate nursing programs, integrating NANDA‐I nursing diagnoses into the curriculum emphasizes students' need to understand the diagnostic process comprehensively. This includes mastering various nursing diagnoses and their contributors (defining characteristics and related factors) in this process. In scholarly discourse, extensive research has scrutinized students' diagnostic competencies, often examining their ability to identify the most frequently utilized NANDA‐I diagnoses. These studies consistently highlight challenges students face, particularly in accurately identifying and applying nursing diagnoses (Ardahan et al. 2019; Basit and Korkmaz 2021; Emine and Asuman 2022). Concurrently, recent investigations have explored diverse teaching methodologies to enhance nursing diagnosis instruction (Abbasi et al. 2017; Basit and Korkmaz 2021; Ordu and Çalışkan 2023). Despite these efforts, limited attention has been given to students' evaluations of these instructional approaches, a critical area for understanding and improving pedagogical effectiveness. Empirical evidence underscores that students represent a particularly discerning source for appraising the productivity, informativeness and satisfaction derived from the learning experience (Theall et al. 2001). Student evaluations are also recognized as significant indicators of teaching quality (Kember and Wong 2000; Safavi et al. 2013; Ulker 2021). Such evaluations offer instructors and institutions essential feedback to tailor teaching methods, addressing students' needs and expectations more effectively. In the short term, these evaluations serve as a vital tool for monitoring and enhancing the quality of education (Ribeiro and Coelho 2019; Saidi and Vu 2021).

## 3 Methods

This study adhered to the Consolidated Criteria for Reporting Qualitative Research (COREQ) guidelines to ensure methodological rigour.

### 3.1 Design

An exploratory, descriptive qualitative design was used to investigate nursing students' evaluations of the instructional approaches employed in nursing diagnosis teaching. This qualitative approach was selected to gain an in‐depth understanding of students' perceptions, experiences and reflections regarding the effectiveness and clarity of nursing diagnosis instruction. The study was guided by a constructivist paradigm, which recognizes that knowledge is co‐constructed through participants' experiences and the researcher's interpretation (Lincoln and Guba 1985).

### 3.2 Sample and Recruitment

Participants were recruited using a nonrandomized snowball sampling method facilitated through various social media platforms. This approach aimed to achieve maximum variation by including students from diverse public nursing faculties across Turkiye. In Turkiye, NANDA‐I nursing diagnoses are integrated into the nursing curriculum as part of the nursing process, typically taught through traditional methods such as lectures, case‐study discussions and clinical training (Basit and Korkmaz 2021).

The sample consisted of fourth‐year nursing students in their final semester of a baccalaureate nursing program during the 2022–2023 academic year. Participants met the following inclusion criteria: (1) enrollment as a fourth‐year nursing student; (2) completion of a nursing diagnosis course; (3) a minimum of four years of clinical practice experience; and (4) willingness to participate voluntarily. Students who lacked clinical practice experience or had no exposure to NANDA‐I nursing diagnoses in preparing care plans or observational activities were excluded. Study recruitment began in May 2023 and continued until data saturation was achieved in June 2023. Recruitment efforts included sharing study information via social media platforms (e.g., Facebook, Instagram, WhatsApp, and email). Faculty members from various institutions supported the dissemination of study notifications to reach eligible participants. Ultimately, 29 students from eight distinct nursing faculties, meeting the inclusion criteria, were interviewed. Data saturation was reached following these interviews, which was consistent with Fusch and Ness's guidelines (2015).

### 3.3 Data Collection

A semi‐structured interview questionnaire was developed based on a review of relevant literature and the research team's expertise. The questionnaire consisted of seven open‐ended questions to gather information about participants' demographic characteristics (e.g., age, university affiliation) and perceptions of instructional approaches in nursing diagnosis education (see Table 1). A pilot study was conducted on 22 March 2023, with three students to evaluate the interview questions' clarity, relevance, and applicability. The pilot provided valuable insights that confirmed the appropriateness of the questions, refined the interview process, and ensured the credibility of data collection. As no revisions were required, the pilot data were included in the main study, thereby strengthening the credibility and rigour of the research findings (Lincoln and Guba 1985).

**TABLE 1:** Semi‐structured Interview Questionnaire.

University Name: Age Could you please elaborate on the clinical practices you have completed as part of your coursework? Are you currently undergoing training, or have you received training on the diagnosis and utilization of NANDA‐I diagnoses at your university? Is there a specific compulsory or elective course within the nursing undergraduate program that covers this? What teaching methods are employed at your university to instruct students on NANDA‐I diagnoses? Do you believe the current methods and content used in teaching NANDA‐I diagnoses are sufficient? If not, what suggestions do you have for improvement? Can you share your personal experiences using NANDA‐I diagnoses in a practical setting? Please describe your experience preparing a care plan using NANDA‐I diagnoses. Would you like to contribute any additional information or insights?

Individual interviews were conducted via Zoom using the semi‐structured interview questionnaire to facilitate in‐depth discussions. One researcher (SK), trained in qualitative research methods, conducted all interviews. Both researchers involved in the study were female faculty members (an associate professor and a research assistant with a PhD), independent of the participants, and experienced in qualitative research and applying NANDA‐I diagnoses.

Participant recruitment and interviews continued until data saturation was achieved, defined as the point at which no new information emerged (Fusch and Ness 2015). Each participant provided verbal consent at the beginning of the interview, and written informed consent was obtained via email, with participants returning a signed copy to the lead researcher.

Interviews were conducted once per participant, lasting between 25 and 40 min. All sessions were audio‐ and video‐recorded, with the interviewer conducting the sessions from her office to ensure privacy. Verbatim transcription of audio recordings and interview notes was performed after all interviews. Participants were allowed to review their transcripts for accuracy; however, no revisions or additions were requested. To protect confidentiality, all transcribed data were thoroughly de‐identified.

### 3.4 Data Analysis

Thematic content analysis, as outlined by Braun and Clarke's (2006) six‐phase thematic analysis framework, was employed to analyse the qualitative data. Interview transcriptions and field notes were organized using MAXQDA20 software to facilitate systematic data management and coding. Each interview audio recording was carefully reviewed, transcribed verbatim, and cross‐checked to ensure accuracy.

The analysis began with a thorough reading of the transcripts, during which both researchers highlighted similar ideas and word groupings and assigned preliminary codes. Both researchers independently read the transcripts multiple times to ensure familiarity with the data and to generate initial codes. Coding was conducted inductively, focusing on recurring patterns and meanings derived from participants' experiences. This process involved iterative discussions between the first and second authors to compare and refine codes, which were then organized into categories. Quotations were selected to illustrate and support the descriptions and enhance the data's trustworthiness.

The transcripts were revisited multiple times, and codes were consistently checked against the original data to ensure credibility and contextual alignment. Codes were grouped into categories and subsequently organized into themes and subthemes through continuous comparison and discussion. The researchers identified emerging themes and subthemes through joint discussions on the final thematic structure. The process of analysis continued until no new themes or subthemes emerged, indicating data saturation.

To enhance rigour, analytic memos and an audit trail documenting coding decisions were maintained throughout the analysis. These procedures supported the dependability and confirmability of the findings (Lincoln and Guba 1985).

The final thematic structure was collaboratively developed and verified against the original transcripts to ensure consistency with participants' narratives.

Rigour and Trustworthiness.

Methodological rigour was ensured following Lincoln and Guba's (1985) criteria of credibility, dependability, confirmability, and transferability. Credibility was supported through pilot testing of the interview questions to ensure clarity, relevance, and appropriateness, independent coding by both researchers, participant transcript verification and peer debriefing with two qualitative research experts.

Dependability was maintained by applying Braun and Clarke's six‐phase framework consistently and documenting analytic decisions, memos, and reflections in an audit trail. Confirmability was reinforced through reflexive journaling by the first author to record assumptions and methodological choices, ensuring that findings remained grounded in participants' perspectives.

Both researchers had prior experience in teaching nursing diagnosis and were familiar with NANDA‐I terminology. To minimize potential bias arising from their professional background, reflexive journaling and regular discussions were conducted throughout the analysis process. Additionally, the researchers had no prior educational or hierarchical relationship with participants, which helped reduce power imbalance and supported open sharing of experiences.

Transferability was strengthened by recruiting 29 students from eight distinct nursing faculties, representing diverse educational contexts and experiences, thereby providing a rich contextual basis for interpretation.

Collectively, these strategies enhanced transparency, analytic rigour, and the overall trustworthiness of the study findings.

### 3.5 Ethical Considerations

Ethical approval for the study was obtained from the Faculty of Ethics Committee at Hacettepe (approval date: 21/03/2023, GO 22/1320). All participants were informed about the study's purpose. Participation was voluntary, and students were included only after providing oral and written informed consent. Data confidentiality and security were rigorously maintained. Audio recordings and written materials were stored as password‐protected electronic files, accessible solely to the first author. These measures ensured compliance with ethical standards and safeguarded the privacy of all participants.

## 4 Results

### 4.1 Participants

The participants, aged between 21 and 23 years, comprised 25 females and four males. All participants had prior experience utilizing NANDA‐I diagnoses during clinical practice, particularly within courses such as Internal Medicine Nursing and Surgical Diseases Nursing.

### 4.2 Identified Themes

Data analysis revealed four overarching themes and four subthemes, representing students' evaluations of instructional approaches: (1) Teaching Methods (Subthemes: Insufficient practical training in clinical settings, Ineffective feedback in clinical practice); (2) Utilization of NANDA‐I Diagnoses in Practical Experience (Subthemes: Challenges encountered in utilizing NANDA‐I diagnoses, Perceptions of suboptimal implementation of nursing diagnoses by nursing professionals); (3) Enhancing Effectiveness: Recommendations for Improving the Teaching of Nursing Diagnosis; and (4) Nursing Students' Perceptions of NANDA‐I Diagnoses (see Table 2).

**TABLE 2:** Themes, Subthemes and Key Findings.

Themes	Subthemes	Key findings
Teaching Methods	Insufficient practical training in clinical settings	Although theoretical instruction was considered adequate, students emphasized a lack of opportunities to apply NANDA‐I diagnoses in real clinical settings and reported inconsistencies in clinical teaching across departments.
	Ineffective feedback in clinical practice	Students reported variability in feedback quality due to overcrowded clinical groups, limited instructor availability, and inconsistent feedback practices among instructors.
Utilization of NANDA‐I Diagnoses in Practical Experience	Challenges encountered in utilizing NANDA‐I diagnoses	Students experienced difficulty linking patient data with appropriate diagnoses, relied on a limited set of familiar diagnoses, and reported insufficient exposure to diverse NANDA‐I domains.
	Perceptions of suboptimal implementation by nursing professionals	Students observed that nurses often use routine, non‐individualized diagnoses, predominantly focusing on physiological needs while neglecting psychosocial and holistic aspects of care.
Enhancing Effectiveness: Recommendations for Improving the Teaching of Nursing Diagnosis	—	Students recommended increasing practice‐based learning, incorporating bedside teaching, improving collaboration with nurses, ensuring consistency among instructors, reducing group sizes, and fostering critical thinking skills.
Nursing Students' Perceptions of NANDA‐I Diagnoses	—	Students acknowledged the importance of NANDA‐I diagnoses for professional communication and care planning but reported difficulties in understanding, cultural adaptation, and practical application, along with concerns about future workload.

### 4.3 Teaching Methods

Participants reported that nursing diagnoses were taught through both theoretical and practical dimensions. The theoretical component introduced students to the types and components of nursing diagnoses and reinforced these concepts through case studies that required application. The practical component involved preparing care plans for patients in clinical settings, complemented by oral or written feedback, depending on the instructor's approach.

Students first encountered nursing diagnoses in the Fundamentals of Nursing Course during their first year. While theoretical instruction emphasized the nursing process in this initial course, students noted diminished emphasis on theoretical content in subsequent years. Instead, they were expected to transfer foundational knowledge into care planning during later classes.

### 4.4 Student Perspectives Illustrate These Observations

- “Only in the Fundamentals of Nursing course were NANDA‐I diagnoses explained, and we were expected to use them in other professional courses as learned.” (Student 3)
- “Since NANDA‐I diagnoses are taught in the first year, instructors assume we already know them in subsequent years.” (Student 1)

#### 4.4.1 Insufficient Practical Training in Clinical Settings

While participants expressed satisfaction with theoretical instruction, they consistently reported insufficient clinical teaching opportunities and highlighted inconsistencies in clinical teaching across departments.

- “Diagnoses are taught theoretically, but there is limited opportunity for implementation in practice.” (Student 6)
- “Although theoretical instruction covers NANDA‐I diagnoses comprehensively, we lack opportunities to apply them in clinical settings.” (Student 11)

#### 4.4.2 Ineffective Feedback in Clinical Practice

Students identified several barriers to effective feedback, including overcrowded clinical groups, limited instructor availability, and inadequate patient numbers for teaching.

- “The clinics are overcrowded, and the student‐to‐instructor ratio limits one‐on‐one discussions with clinical instructors.” (Student 9)
- “There are variations in feedback quality—some instructors provide immediate feedback, while others delay or omit it altogether.” (Student 14)

### 4.5 Utilization of NANDA‐I Diagnoses in Practical Experience

Students' application of NANDA‐I diagnoses was primarily limited to preparing care plans during clinical practice.

- “We are expected to develop a care plan using NANDA‐I diagnoses for the patients under our care in clinical settings.” (Student 10)

#### 4.5.1 Challenges Encountered in Utilizing NANDA‐I Diagnoses

Students expressed difficulties linking patient data with appropriate NANDA‐I diagnoses, often relying on a limited set of diagnoses due to a lack of confidence and exposure.

- “We frequently use the same diagnoses because we're unsure about interpreting the data or lack experience incorporating it into care plans.” (Student 1)
- “Our focus tends to be on general physiological needs, neglecting domains like family processes, sexuality, and values.” (Student 4)
- “While the diagnosis books highlight over 200 NANDA‐I diagnoses, our consistent reliance on a limited set of diagnoses raises questions about the distinctions between them and the criteria for selecting a diagnosis based on available data in the educational context.” (Student 5)

Furthermore, students expressed a need for more experience in applying diagnoses across various domains of NANDA‐I in both theoretical and clinical contexts. Despite the extensive array of over 200 NANDA‐I diagnoses, participants reported consistently identifying only 7 or 8 diagnoses as care problems during their training, with limited exposure to diagnoses in different domains. Students also observed a similar trend in clinical practice, noting that nurses tended to focus on a narrow range of NANDA‐I diagnoses.

- “Frequently used diagnoses include risk for fall, risk for infection, pain, and risk for bleeding, disturbed sleep pattern, bathing self‐care deficit, and disturbed body image. I have not encountered any diagnoses related to sexuality (sexual dysfunction, reproduction) and life principles (values, beliefs, moral distress).” (Student 8)
- “Diagnoses that are familiar provide a comfort zone and a sense of security. The prospect of utilizing unfamiliar diagnoses that I have not encountered before induces anxiety and concern about potential errors.” (Student 6)

#### 4.5.2 Perceptions of Suboptimal Implementation of Nursing Diagnoses by Nursing Professionals

Students articulated their observations of nursing care plans prepared by nurses in clinical practice, noting deficiencies in applying nursing diagnoses. According to the students, nurses predominantly compose care plans electronically or in paper format, but the diagnoses delineated in these plans need to be revised. Specifically, there is a tendency to overlook the biopsychosocial aspects of patients, with an overemphasis on the physiological domain, resulting in a lack of holistic patient care evaluation.

- “In my observations, nurses utilize NANDA‐I diagnoses in computer‐integrated care plans and paper‐based formats. However, nurses tend to employ a restricted set of stereotypical diagnoses (such as commonly used diagnoses like pain, self‐care deficit, disturbed sleep pattern, anxiety, knowledge deficiency, and risk for falls), mirroring the patterns observed among us students. Notably, these diagnoses lack individualization. For instance, the risk for fall diagnosis is assigned based on the same data set for all patients, irrespective of unique patient circumstances. Similarly, the preventive measures for addressing the risk of falling also exhibit uniformity. Patients are not actively involved in the care planning process; instead, decisions are made for them without their direct input.” (Student 10)
- “Diagnoses are transcribed into electronic records, with identical care plans and diagnoses assigned to each patient, disregarding individual nuances.” (Student 9)

### 4.6 Enhancing Effectiveness: Recommendations for Improving the Teaching of Nursing Diagnosis

To support the efficacy of teaching the subject, students proffered suggestions to foster consistency among instructors and enhance practical instruction through collaborative bedside discussions with instructors and cooperation with nurses. Selected direct statements from students include:

- “The subject cannot be adequately mastered within the confines of a single course; it merits distinct placement in the curriculum, preferably as an elective or a dedicated diagnostic course.” (Student 8)
- “Teaching should extend beyond theoretical transfer, incorporating practical bedside instruction with instructors during clinical practice. Diagnosis determination should be a collaborative process at the bedside.” (Student 13)
- “Opportunities should be created for joint care plan preparation with nurses during clinical practice, offering students valuable experiential learning.” (Student 17)
- “Uniform face‐to‐face feedback from all instructors across departments is advocated, emphasizing constructive feedback rather than assignment‐based evaluations.” (Student 14)
- “Clinical practice group sizes should be reduced, or the availability of instructors should align with the number of students for a more personalized learning experience.” (Student 5)
- “Training sessions on the identification and utilization of NANDA‐I diagnostic resources should be provided.” (Student 15)
- “Given the analytical and critical thinking demands of the subject, instructional methods should be tailored to develop critical thinking skills among students.” (Student 13)
- “NANDA‐I diagnoses should be systematically presented, associated with the patient's medical diagnosis for enhanced comprehension.” (Student 9)

### 4.7 Nursing Students' Perceptions of NANDA‐I Diagnoses

Students assert a heightened awareness of NANDA‐I diagnoses, emphasizing their belief in incorporating them into nursing practice. However, they acknowledge encountering challenges in utilizing specific diagnoses due to perceived ambiguity and the anticipated intensity of clinical workload post‐graduation. Direct statements from students include:

- “While I am certain that I will integrate NANDA‐I diagnoses into my practice post‐graduation and recognize their significance, I find them somewhat sophisticated. In the Turkish context, the meanings and expressions often elude me. There is a need for a translation that aligns with our cultural perspective.” (Student 10)
- “I firmly believe that NANDA‐I diagnoses should be employed to enhance professional visibility and facilitate common communication through a shared language.” (Student 1)
- “Despite routinely planning patient care needs upon the initial encounter, nurses, in practice, often neglect to document these needs using NANDA‐I diagnoses. The formal recording of these diagnoses is not a prevalent practice.” (Student 2)

## 5 Discussion

This study explored nursing students' evaluations of instructional approaches used in teaching nursing diagnosis. Consistent with previous studies on nursing diagnosis education, the findings suggest that although theoretical instruction was perceived as adequate, important gaps remain in clinical application, feedback processes, and exposure to diverse NANDA‐I domains (Ardahan et al. 2019; Korkut et al. 2021).

### 5.1 Teaching Methods

While students acknowledged the adequacy of theoretical instruction, dissatisfaction was expressed regarding the clinical teaching component. Similarly, Korkut et al. (2021) reported that short clinical practice durations and frequent patient rotations hinder students' ability to implement care plans, an essential aspect of nursing education. Although students demonstrated the capacity to translate theoretical knowledge into clinical practice, the complexity of formulating nursing diagnoses, including data collection, interpretation, clustering, and labeling, requires more robust practical training in authentic clinical settings (Bourbonnais and Kerr 2007).

Although conventional lecture methods are useful for explaining theoretical concepts, they may be insufficient for supporting practical application; therefore, recent literature recommends innovative teaching approaches such as virtual gaming simulation (Ordu and Çalışkan 2023), web‐based mind maps (Ordu and Caliskan 2022), and web‐based teaching (Basit and Korkmaz 2021). Students reported irregular, delayed, and ineffective feedback during clinical practice. Similar findings were reported by Farzi et al. (2018) and Korkut et al. (2021), who highlighted challenges in obtaining consistent feedback. Wong and Shorey (2022) also identified dissatisfaction with feedback quality and quantity. Feedback plays a critical role in developing clinical competence by providing guidance, supporting reflection, and reinforcing appropriate practices (Adamson et al. 2018; Ahea et al. 2016; Burgess and Mellis 2015; Chowdhury and Kalu 2004; Henderson et al. 2019). Barriers such as limited time, communication challenges, and insufficient instructor availability have been reported in previous studies (Alfehaid et al. 2018). Our findings mirror these challenges, particularly in overcrowded clinical groups. Therefore, establishing structured feedback mechanisms and improving instructor availability may enhance students' clinical learning experiences.

### 5.2 Utilization of NANDA‐I Diagnoses in Practical Experience

The second central theme, the utilization of NANDA‐I diagnoses in practical experience, highlights the importance of understanding the taxonomy, including domains, classes, and diagnoses, for achieving program objectives (Herdman et al. 2021). However, students reported difficulties in identifying diagnoses across diverse domains. Previous studies similarly indicate that students tend to focus on a limited set of commonly used diagnoses, while domains such as sexuality, coping/stress tolerance, self‐perception, and role relationships are often overlooked (Ardahan et al. 2019; Emine and Asuman 2022; Noh and Lee 2015; Park and Jeong 2022). These challenges may stem from limited proficiency in data collection and interviewing skills, which can hinder accurate diagnosis formulation (Du et al. 2022; Emine and Asuman 2022).

Consistent with earlier research, students in this study reported insufficiencies in data collection during clinical practice despite being equipped with semi‐structured forms. These findings suggest a need for additional support in comprehensive data gathering, including guidance on initiating conversations, identifying appropriate timing for inquiries, and establishing patient trust. Students also recommended integrating discussions of at least one diagnosis from each of the 13 domains or 47 classes of the NANDA‐I taxonomy into both theoretical and clinical education to broaden exposure and enhance understanding of the taxonomy.

Although clinical nurses provided support during practice, many students expressed concerns regarding their adequacy as role models. Reported limitations included insufficient holistic patient assessment, a predominant focus on physiological problems, and limited attention to other domains in care plans. Similar concerns were reported by Karadağ et al. (2013), who identified inadequate role‐model behaviour among clinical nurses. Türen et al. (2017) highlighted deficiencies in nursing care plans, while Atakro et al. (2019) emphasized that the abstract nature of nursing diagnoses and limited use of care plans in clinical practice create challenges for students (Atakro et al. 2019; Türen et al. 2017).

Observing clinical nurses plays a significant role in students' professional development; consistent with Bandura's learning theory, students acquire knowledge through observation, making clinical nurses key facilitators in the educational process (Bandura 2001; Raso et al. 2019).

### 5.3 Enhancing Effectiveness

Recommendations for Teaching Nursing Diagnosis.

Students emphasized the need for increased clinical support, particularly through bedside instruction, collaborative care planning with nurses, and consistent departmental feedback. Improving clinical education may require smaller student groups, greater instructor availability, and the integration of structured teaching strategies to enhance critical thinking and diagnostic reasoning skills (Berndtsson et al. 2020). Additionally, offering elective courses or dedicated sessions on nursing diagnosis may provide students with opportunities for deeper learning and skill development.

Nursing Students' Perceptions of NANDA‐I Diagnoses.

Despite challenges in learning and applying NANDA‐I diagnoses, students emphasized their importance in establishing a common professional language in nursing. However, concerns related to workload intensity and the perceived cultural incompatibility of certain NANDA‐I terms indicate the need for contextual adaptation and additional educational support during the learning process.

### 5.4 Limitation

This qualitative study was conducted in eight public nursing schools within a single country and included a limited sample of volunteer participants. Therefore, the findings may not be generalizable to other nursing schools or cultural contexts. Future research should involve larger and more diverse samples and examine cross‐cultural perspectives on teaching nursing diagnosis.

Additionally, the use of snowball sampling may have introduced selection bias, as participants were recruited through professional networks and social media, potentially favouring students who were more engaged with or interested in NANDA‐I diagnoses. Furthermore, the digital nature of data collection via Zoom may have influenced the depth and richness of responses compared with face‐to‐face interviews, particularly by limiting opportunities for contextual observation and non‐verbal communication.

Despite these limitations, the study provides valuable insights into nursing students' perspectives on the teaching of NANDA‐I nursing diagnoses in Türkiye. The findings offer guidance for future research, nursing education, and curriculum development by highlighting areas where targeted educational strategies may enhance students' understanding, application, and consistent use of standardized nursing language in clinical practice.

## 6 Conclusion and Recommendations

This study explored fourth‐year nursing students' perceptions of teaching approaches for nursing diagnosis. Although theoretical instruction on NANDA‐I was perceived as comprehensive, students reported insufficient practical training and limited timely feedback in clinical settings. Participants also indicated that clinical nurses did not consistently address care problems across all NANDA‐I domains.

Students emphasized the importance of interactive bedside teaching, increased opportunities to practice NANDA‐I diagnoses, and timely feedback on care plans. The identified gap between theoretical knowledge and clinical application highlights the need for strengthened practice‐based learning opportunities prior to graduation.

Recommendations:

- Review and strengthen the clinical teaching framework to better align with student learning needs.
- Increase structured practical training opportunities to support the development of diagnostic skills.
- Promote interactive teaching methods, particularly bedside discussions and collaborative learning.
- Provide timely and constructive feedback to enhance student learning.
- Ensure exposure to a broader range of NANDA‐I diagnoses in both theoretical and clinical education
- Encourage clinical nurses to serve as positive role models in the use of standardized nursing diagnoses.
- Integrate innovative teaching approaches, such as simulation, alongside traditional instructional methods.

## Author Contributions

**Sıdıka Kestel:** conceptualisation, methodology, validation, data collection, writing, original draft, writing. **Fatoş Korkmaz:** conceptualisation, methodology, validation, reviewing and editing, and supervision. The first author conceived the project. Both authors contributed significantly to the work's design and data acquisition, analysis, and interpretation. Both authors were involved in writing the work and critically revising it for important intellectual content. Both parties have agreed on the final version to be published.

## Funding

The authors have nothing to report.

## Ethics Statement

Written permission was obtained from The Non‐Interventional Clinical Research Ethics Committee of the university in which the study was undertaken (date: 21/03/2023, number: GO 22/1320).

## Conflicts of Interest

The authors declare no conflicts of interest. Personal relationships that could have appeared to influence the work reported in this paper.

## Data Availability

The data that support the findings of this study are available from the corresponding author upon reasonable request.
